# A case report of para-esophageal bronchogenic cyst with esophageal communication

**DOI:** 10.1186/1749-8090-7-94

**Published:** 2012-09-26

**Authors:** Wuping Wang, Yunfeng Ni, LiWang Zhang, Xiaofei Li, Changkang Ke, Qiang Lu, Qingshu Cheng

**Affiliations:** 1 Department of Thoracic Surgery, Tangdu Hospital, The Fourth Military Medical University, Xi'an, PR China; 2 Department of Scientific Research, Tangdu Hospital, The Fourth Military Medical University, Xi'an, PR China

**Keywords:** Bronchogenic cyst, Esophageal cyst, Mediastinal cyst

## Abstract

Paraesophageal bronchogenic cyst was one of common mediastinal congenital cystic lesions of foregut origin. Because of an intimate embryologic relationship with the esophagus, they were usually found intramural (intramural esophageal bronchogenic cysts) with the local esophageal mucosa being intact and the paraesophageal bronchogenic cysts were rarely communicated with esophageal lumen. We report a case of para-esophageal bronchogenic cyst communicating to the esophageal lumen thorough a pedicle of canal, which looked liked a diverticulum on X-ray. During the operation, a communication of paraesophageal bronchogenic cyst with esophageal was found and the pathology diagnosis were made then. The symptoms of chest pain and dysphagia were relieved immediately after operation. The follow-up was well 2 years after the surgery.

## Background

Bronchogenic cysts were congenital lesions derived from the primitive foregut, and were the most common primary cysts of the mediastinum [1,2]. They usually located in close relation to tracheobronchial tree or esophagus for their foregut origin. Among them, para-esophageal bronchogenic cysts were usually closely adherent to esophagus with or without a well-defined borderline [3]. Sometimes the bronchogenic cysts located within the wall of the esophagus with esophageal mucosa intact, which were also called intra-mural esophageal bronchogenic cyst [4,5]. However, the communication of cysts and the esophageal lumen was rarelly found. Previously, only five cases of para-esophageal bronchogenic cysts communicate with esophageal lumen were reported [6-9]. Here, we report a case of an adult with paraesophageal bronchogenic cyst with esophageal communication, who underwent successful surgical treatment.

## Case presentation

**Figure 1:**
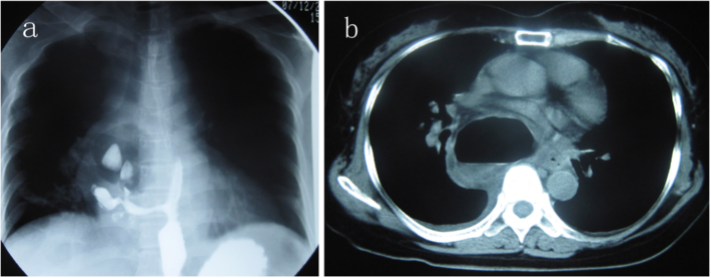
**(a) A barium swallowrevealed a huge pare-esophagealcyst cavity compressed toesophagus,and also communicated toesophagus through a longentrance.** (**b**) The chest CT scans showed a huge cystic mass full of gas and liquid located in the post-mediastinum with a direct compression of the pericardium, esophagus and right lung.

**Figure 2:**
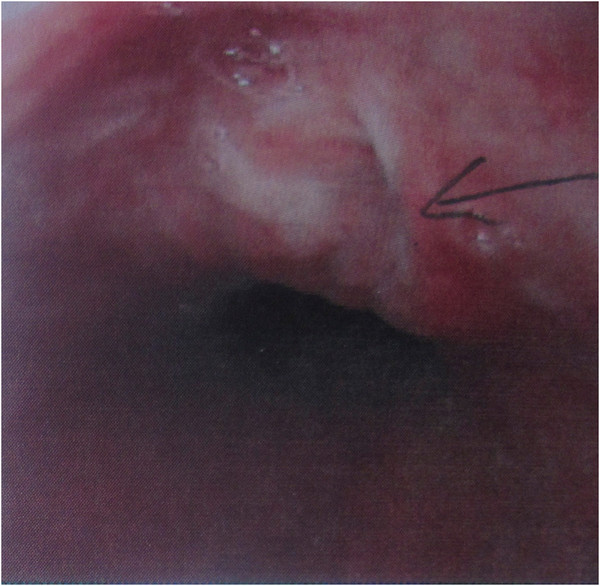
**Uper endoscopy revealed ano ulcero-necrotical entrance (1.5 cm** **× 1.0) on theright anterior wall ofthe esophageal at 36** **cm from scior teeth,where the local mucosaare smooth and thoroughly (seethe arrow).**

**Figure 3:**
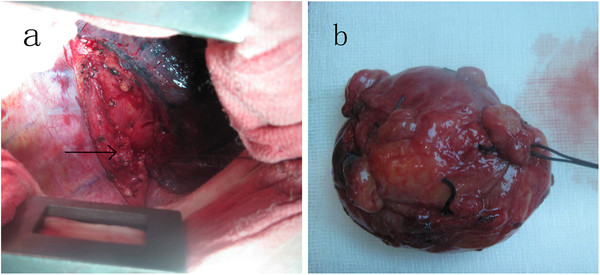
**Complete remove of thepara-esophageal cyst through aright poster-lateral thoractomy.** (**a**) Gross appearance of the resected cyst. (**b**) The entrance on the esophagus was sutured in two layers. The arrow points the pedicle after suture.

**Figure 4:**
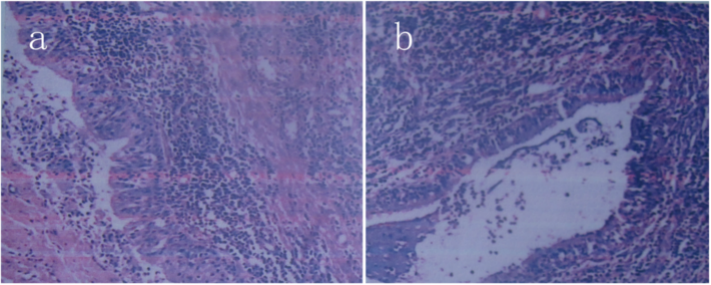
**The pathology examination ofthe cystic wall showedbronchogenic tissue including respiratoryciliated columnar epithelium (a,HE,×100), smooth muscle andCartilage (b, HE,×40)**

A 56 years old woman was admitted into our hospital with symptoms of intermittent and reprieved chest pain and dysphagia over 3 years as well as a rapid weight loss of 10 kg in two months. She has no fever, cough, and shortness of breath. And there was no significant positive finding in physical examination. A barium swallow revealed a huge para-esophageal cyst compressed to esophagus, and also communicated with esophagus through a canal (Figure 1a). The chest computer tomography scan showed a huge cystic mass full of gas and liquid located in the posterior mediastinum with a direct compression of the pericardium, esophagus and right lung (Figure 1b). However, the gastroendoscopy revealed a ulcero-necrotical hole (1.5 cm × 1.0) on the right anterior wall of the esophageal at 36 cm from the incisors, and the local surface mucosa were smooth (Figure 2). So, differential diagnoses included esophageal diverticulum, esophageal fistulas to mediastinum mass, para-esophageal bronchogenic cyst with esophageal communication, a duplication cyst was considered before operation. The patient underwent right posterlateral thoractomy. The cyst was found in situated lower pare-esophageal, segment and communicated to esophagus through a pedicle about 1.3 cm of the width, and 1.2 cm of the length, with an intact inner surface. There was no bronchial communication. The well demarcated and capsulated 8 cm × 7 cm × 7 cm cyst was excised completion (Figure 3a). The opening on the esophageal wall was closed by a 4–0 interrupted suture in whole layers (Figure 3b). The postoperative evolution was uneventful with a rapid and total recovery. Ciliated columnar epithelium lining the cyst (Figure 4a) and cartilage in the walls with smooth muscle (Figure 4b) were found in the postoperative pathology and cyst was confirmed. The symptoms of chest pain and dysphagia were relieved immediately after operation. The follow-up was satisfied 2 years after the surgery.

## Discussion

Bronchogenic cysts were the most common congenital cystic lesions of mediastinum, originated from the primitive foregut. The dorsal segment of the foregut formed the esophagus, while the ventral component formed the trachea [1,2]. The mediastinal bronchogenic cyst was occurred when there was abnormal budding or branching of tracheobronchial tree within early stage of the gestation [1,2,10]. The most common location of bronchogenic cysts in mediastinum includes para-tracheal, sub-carina, hilar, para-esophagus and pericardium [3]. Bronchogenic cysts were also reported in intra-pericardium, pre-sternal, supraclavicular spaces, sub-diaphragmatic, retro-peritoneal and skin [11-15].

For pare-esophageal bronchogenic cysts, the extramural pare-esophageal bronchogenic cyst was the most common type. They were always found anotomic closed to esophagus, with clearly borderline or with only a mind adhesion to gastrointestinal tract, which made the operative procedure difficult. Some cystic lesions tend to located in the wall of esophagus, but with lumen mucosa intact. This type of bronchogenic cysts had been recognized well as intramural esophageal bronchogenic cysts in the literatures [4,5]. Even though the mass was completely embedded in the wall of esophagus, the pathology showing hyaline cartilage and respiratory epithelium in the wall of cystic lesions,which confirmed its bronchogenic origin [4,5,16].

In our case, here we emphasized a more rare type of para-esophageal bronchogenic cyst, in which the cystic lesion communicated to esophageal lumen through a prepared canal. To our knowledge, only five similar case of bronchogenic cyst had ever been reported, but the nation of the communication remained unclear [6-9]. The main controversial point was whether the communication existed congenitally or it was formed posteriorly. In 1978, Mindelzun R and his colleagues first reported a mediastinal bronchogenic cyst with esophageal communication. They believed that thought the surrounding inflammation made it impossible either surgically or pathologically to establish the exact etiology of the fistula formation [6]. So whether this represents a post-inflammatory change or a congenital communication remained a matter of conjecture. In 1999 when Knezevic J and his colleagues present a case of a well prepared para-esophageal bronchogenic cyst connected with the esophageal lumen by a narrow canal composed of stratifed squamous epithelium [8]. In this patient, the transition of the ciliated epithelium of the cyst into the stratifed squamous epithelium of the cystic canal serves to emphasize the close anatomic and embryologic relationship between esophagus and the bronchial cystic derivatives [8]. Consider the context of their embryologic development from the foregut, as well as on a histologic basis, the presence of an esophageal communication was thought less remarkable. Our case was similar with Knezević J's report [7]. We also found a well prepared cyst connected to esophageal lumen through a canal, on which the inner face of the canal was covered by intact sequential mucosa. This rare type might be classified as lumen-communicated pare-esophageal bronchogenic cysts, which should not be confused with fistula between a mediastinal cysts and esophagus due to inflammatory invasion. The latter are often characterised by ulcerative change under endoscopy with mucosa destroy, confirmed by Olivier N. Pages and his colleague, In their report, an ulcero-necrotic esophageal wall over a 3 cm^2^ zone was found and a fistula of the bronchogenic cyst into the esophagus was revealed in video-assisted thoracoscopic surgery in emergency revealed [9]. Instead of an intact inner face of canal confirmed in Knezević J's study and our case research. In our opinion, a lumen-communicated pare-esophageal cyst should include some teatures. First, a well prepared pare-esophageal cystic lesion communicated to esophageal lumen. Second, the communication happened through complete canal. Third, the inner face of the canal was covered by intact sequential mucosa characterized by respiratory epithelium to enteric lining. The fourth, there was no evidence of fistula, and there was a non-ulcerative appearance under endoscopy, which are similar with esophageal diverticulum. Although the incidence of communication between the esophagus and the bronchial cystic derivatives has not been established, from the 6 cases include ours reported, we should considered the existence of this rare type of bronchogenic cysts.

The clinical symptoms of patients with bronchogenic cysts were varied according to the lesions' size, location and communication. For the Pare-esophageal cysts, the patient more likely present symptoms of dysphagia and chest pain due to compression and pleural irritation [1,3,4]. But for the lumen-communicated pare-esophageal cyst, the symptoms may also associated with the penetration of liquid and air through the canal from the esophagus into the cyst [8]. This can explain why our patient present with an irreversible dysphagia and chest pain in his past history. However, Infection was no found in our case.

For a pare-esophageal cyst, an accurate pre-operative diagnosis was difficult. Classic imaging features clould only infer the possible of lesion. Although accurate diagnosis must be provided by postoperative pathology, and per-operative differentiation should include other cystic lesions around esophagus, such as esophageal duplication cyst, esophageal malioloma or mediastinal tumor. In case of difficult preoperative diagnosis of a para-esophageal mass, endoscopic ultrasound was a safe and very useful procedure to clearly distinguish cystic from solid masses as well as defining the intraextramural extent of the lesion [17]. In our case, we focused on the differentiation with a diverticulum if communication was of coexistence.

All suspected bronchogenic cysts were suggested to be removed in operable candidates. Because complete surgical resection would contribute to establish diagnosis, alleviate symptoms, and prevent complications [10]. We considered the connection in our case was a congential communication, instead of fistula formation. The operation procedures were simple and it's unnecessary to suture the mucosa, and submucosa tissue, respectivelly. Full-layer suture of the neck of canal along esophageal wall in twice was enough for a safe repairment. Embrace with omentum or chest pleural are unnecessary, which may be suitable in suspected individual with severe infection [9,18].

## Conclusion

In conclusion, the lumen communicated para-esophageal bronchogenic cyst may be a rare congenital cystic lesion of mediastinum. More cases should be reported to confirm the nature of the communication.

## Consent

Written informed consent was obtained from the patient for publication of this Case report and any accompanying images. A copy of the written consent is available for review by the Editor-in-Chief of this journal.

## Competing interests

The authors declare that they have no competing interests.

## Authors' contributions

WW, study concept and design; YN, acquisition of data; analysis and interpretation of data; LWZ and CK, critical revision of the manuscript for important intellectual content and material support; XL, technical, or material support; QL, drafting of the manuscript; QC, drafting of the manuscript. All authors read and approved the final manuscript.

## Authors' information

Wuping Wang, bachelor's degree, Superintendent of the institution, surgeon; Yunfeng Ni, doctor's degree, surgeon; Changkang Ke, master's degree, surgeon; Xiaofei Li, doctor's degree, surgeon; Qiang Lu, doctor's degree, surgeon; Qingshu Cheng, doctor's degree, surgeon;
